# Optimal low voltage ride through of wind turbine doubly fed induction generator based on bonobo optimization algorithm

**DOI:** 10.1038/s41598-023-34240-6

**Published:** 2023-05-13

**Authors:** M. Abdelateef Mostafa, Enas A. El-Hay, Mahmoud M. Elkholy

**Affiliations:** Electrical Power and Machines Engineering Department, Faculty of Engineering, Zagazig University, Zagazig, Egypt

**Keywords:** Engineering, Electrical and electronic engineering

## Abstract

The large-scale wind energy conversion system (WECS) based on a doubly fed induction generator (DFIG) has gained popularity in recent years because of its various economic and technical merits. The fast integration of WECS with existing power grids has caused negative influence on the stability and reliability of power systems. Grid voltage sags produce a high overcurrent in the DFIG rotor circuit. Such these challenges emphasise the necessity of the low voltage ride through (LVRT) capability of a DFIG for ensuring power grid stability during voltage dips. To deal with these issues simultaneously, this paper aims to obtain the optimal values of injected rotor phase voltage for DFIG and wind turbine pitch angles for all operating wind speeds in order to achieve LVRT capability. Bonobo optimizer (BO) is a new optimization algorithm that is applied to crop the optimum values of injected rotor phase voltage for DFIG and wind turbine pitch angles. These optimal values provide the maximum possible DFIG mechanical power to guarantee rotor and stator currents do not exceed the rated values and also deliver the maximum reactive power for supporting grid voltage during faults. The ideal power curve of a 2.4 MW wind turbine has been estimated to get the allowable maximum wind power for all wind speeds. To validate the results accuracy, the BO results are compared to two other optimization algorithms: particle swarm optimizer and driving training optimizer. Adaptive neuro fuzzy inference system is employed as an adaptive controller for the prediction of the values of rotor voltage and wind turbine pitch angle for any stator voltage dip and any wind speed.

## Introduction

Currently, wind energy is regarded as one of the renewable resources with the fastest rate of growth and the most attractive renewable energy all over the world due to its high power density and large availability^1–3^. Wind energy contributed for 3.5% of the international demand of electricity in 2011, and this percentage is expected to increase to 16% in 2030^4^. Nevertheless, the integration of WECS into the grid is still a major challenge because of power quality issues, the intermittent nature of wind, resonance, capacitor switching, etc. Amongst power quality issues, the unexpected dip in the terminal voltage of wind generator because of a fault on the grid side may cause wind turbines to trip suddenly from the grid^5,6^. The stability of grid may be adversely impacted by wind generators that trip and reconnect frequently. Many countries have developed new grid codes for grid-connected WECS in order to reduce the wind generators tripping from the grid and to keep the grid stable in the event of faults or grid voltage abnormalities^7–10^.

DFIGs are the most efficient option for WECSs due to their significant benefits over other wind generator types^11^. The benefits of DFIG include controllable ability for both active and reactive power, small size, the necessity of lower rated converters, which results in lower power losses and converter cost, acoustic noise and mechanical stress reduction, variable speed generation, and power quality enhancement^12–14^. On the other hand, the major disadvantage of using a DFIG in WECS is its sensitivity to grid disturbances, particularly voltage dips, because the DFIG stator terminals are directly linked to the grid. The sags in grid voltage produce a large overcurrent in the DFIG rotor circuit. As a result, it leads to activating the circuits of protection and DFIG is disconnected from the grid for protecting the rotor-side converter^15–17^.

LVRT capability is the most prevalent grid code requirement. It is the ability to stay connected to the grid during voltage sags and also deliver reactive power to support grid voltage in the event of faults^6,18,19^. Dips in voltage are very critical disturbances for DFIG. These sags lead to an increase in stator and rotor currents, so the power that can be injected into DFIG must be reduced^20–22^. According to several literature surveys, pitch control method, hardware methods, and modified DFIG converter control method are the improvement of LVRT strategies^12,23,24^. For the pitch control method of LVRT improvement, the power of wind turbine can be lowered by adjusting the pitch angle of rotor blades. Nevertheless, because of the slow mechanical dynamics, this technique performs poorly^25^. Crowbar protection and energy storage system are two main categories of hardware methods for LVRT improvement^26–29^. The basic idea of crowbar method is to activate a resistor bank in the circuit of DFIG rotor in the event of faults, which results in extra power drain and limit DFIG currents. This method has several glaring problems, including high stress on drive train caused by electromagnetic torque fluctuations, controllability loss, and preventing the grid voltage recovery because of absorbing of reactive power. While the other type of hardware methods for enhancing LVRT is employing a type of energy storage system like battery energy storage system, flywheel energy storage system, electrical double-layer capacitor, and superconducting magnetic energy storage^5,21,23,30–32^. But, the major drawback of this method is not an economical solution. Because of the disadvantages of pitch control and hardware methods, the LVRT capability can be improved by modifying of DFIG converter control which represents in changing the reference control values in DFIG converter control^24^. The modified DFIG converter control method is the most cost-effective technique for LVRT improvement owing to its advantages like ease of implementation, lower cost, easy switching back to normal operation, and DFIG is always under control^30,33–35^.

The recent modified control approaches have been suggested in recent literature to enhance LVRT capability for DFIG based WECS. In reference^20^, A. Tilli et al. introduced a novel modified control for a back-to-back converter based on nonlinear control theory arguments. The proposed solution adopts both feedback and feedforward terms for avoiding rotor overcurrents to prevent rotor-side converter tripping. The advantages of proposed method are robustness, providing additional oscillation damping, and minimal assistance of additional protective hardware. In reference^36^, M. A. S. Ali et al. presented a modified control for the rotor converter to suppress DC link voltage fluctuations and rotor overcurrents for improving LVRT capability. The references of rotor voltage are injected with additional voltage terms for enhancing the dynamic behaviour of DFIG based WECS. There is no effect on the stability of the current loops since the voltage terms are introduced outside of them. Moreover, the electromagnetic torque fluctuations that occur during faults are greatly reduced. In reference^4^, M. K. Senapati et al. proposed a modified demagnetization control method for improving LVRT capability for DFIG in the case of grid faults. By employing demagnetization control and an external resistance on the DFIG stator side, the proposed control technique is accomplished in a coordinated way. The LVRT improvement can be achieved by damping the dc component of stator flux by demagnetization control and accelerating transient flux damping by the external resistance. The major merits of this proposed method are better dynamic responses, the security operations improvement, power system stability enhancement, and achieving LVRT capability with the higher penetration of wind energy. In reference^34^, G. Manohar et al. presented a hybrid approach-based control model for improving LVRT capability of DFIG based WECS. The hybrid method combines the execution of the random forest algorithm and the modified elephant herding algorithm. The optimal solutions from the available searching space and the creation of training dataset are identified offline by the modified elephant herding algorithm which takes into consideration multiple parameters which related to LVRT like, current, voltage, and active and reactive powers. In reference^37^, R. Hiremath and T. Moger introduced a modified super-twisting algorithm for the LVRT improvement under voltage dip condition. The proposed method employs the second-order sliding mode for controlling the DFIG based WECS. It was found that the proposed method improved LVRT capability for the single wind turbine DFIG system and the practical wind farm under transient conditions. In reference^38^, G. Manohar et al. proposed a hybrid system based on fertile field algorithm and momentum search algorithm with ANFIS to enhance LVRT capability of DFIG based WECS. The optimization technique is employed to solve the objective function that is related to LVRT. The better probable control signals for rotor-side and grid-side converters are performed and forecasted by ANFIS. The proposed method can overcome the problem of voltage and system instability as well as improve the LVRT capability. In reference^39^, A. Chakraborty and T. Maity presented a novel application of adaptive fuzzy logic controller for LVRT improvement of DFIG based WECS. A cascaded adaptive fuzzy logic control is employed to adjust the rotor-side and grid-side converters to enhance the DFIG based WECS performance. The proposed methods can feed the smooth reactive and active power to the grid during severe disturbances resulting in LVRT enhancement.

A wide variety of optimization techniques are proposed to solve a wide range of complicated engineering problems, which are frequently non-linear and non-continuous problems. The main advantages of these algorithms in comparison to mathematical techniques are easy implementation, flexibility, robustness, and computational efficiency^40–43^. ANFIS controllers are used to control multi-input, single-output, and non-linear systems. The ANFIS controller has the advantages of both fuzzy logic and neural networks. The key benefits of implementing ANFIS controllers are performance enhancement, no need for a system mathematical model, the design depends on real system data, and need less effort to tune^25,28,44–50^.

From the literature review, it is clear the importance of obtaining the reference values of rotor voltages that ensure preventing rotor over currents during grid voltages dips to improve LVRT capability. Therefore, this paper presents additional contributions in this research point which can be summarized as follow: (i) Obtaining the optimal values of rotor voltages and pitch angle for a wide range of wind speed from 4 m/s to 25 m/s and at different values of stator voltages, (ii). These optimal values ensures that the DFIG develops the allowable maximum power without exceeding the rated values of stator and rotor currents, (iii) Application of BO and DTA as novel optimizers which are developed in 2022 to crop the optimal values of rotor voltages and pitch angle at different wind speeds and stator voltages, and (iv) developing an ANFIS controller to ensure fast prediction to the optimal rotor voltages and pitch angle for any stator voltage and wind speed. The BO results are verified by two other optimization methods; one of them is well-known optimization algorithm (PSO) which is presented in detail in reference^51^.

The rest of this paper is structured as follows. The wind turbine aerodynamic model is introduced in Section “Wind turbine aerodynamics model” to get the values of wind turbine pitch angles for all operating wind speeds where, these values provide maximum wind turbine power values which ensure that the DFIG stator and rotor currents do not exceed their rated values. In Section “DFIG steady-state model”, the electrical steady-state model of DFIG considering iron losses is described to get the values of injected rotor phase voltage for DFIG for achieving LVRT capability with providing the maximum reactive power to the grid for supporting grid voltage during voltage dips. While, Section “Bonobo optimizer” demonstrates a description of the BO that is used to achieve these goals by cropping the optimal values of rotor phase voltage for DFIG by using the steady-state DFIG equations and wind turbine pitch angles by using wind turbine equations. The results of the reference values of injected rotor voltage and pitch angles to achieve LVRT capability are presented in Section “Results and discussion”. Then, Section “ANFIS controller” introduces the proposed controller based on ANFIS to predict the values of rotor voltage and pitch angle for any stator voltage dip and any wind speed. Lastly, Section “Conclusion” concludes this paper.

## Wind turbine aerodynamics model

**Figure 1:**
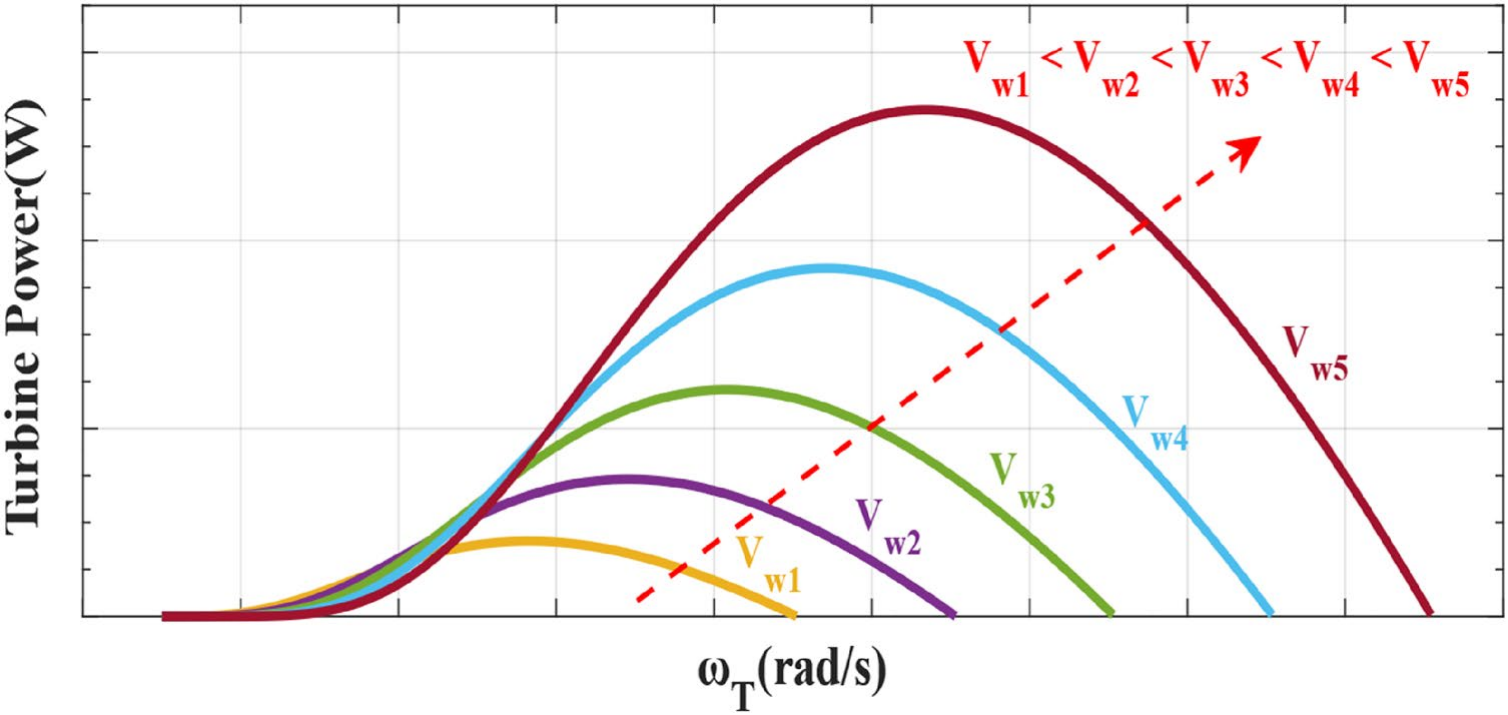
The wind turbine power with rotational speed under various wind speeds.

The greatest possible proportion of available captured air power by a wind turbine is 59.26% in accordance with Betz’s law^52^. A wind turbine is used to capture the kinetic energy from wind and convert it into mechanical power that is used to drive the wind generator. The model of wind turbine is given as follows^53–56^:

1 $${P}_{turbine} = {C}_{p}\left(\beta ,\lambda \right){P}_{wind}=\frac{1}{2}{C}_{p}\left(\beta ,\lambda \right)\rho \pi {R}^{2}{V}_{w}^{3}$$

2 $${C}_{p}\left(\beta ,\lambda \right)=0.73\left(-13.2-0.58\beta -0.002{\beta }^{2.14}+\frac{151}{{\lambda }_{i}}\right){e}^{\frac{-18.4}{{\lambda }_{i}}}$$

3 $$\frac{1}{{\lambda }_{i}}= \frac{1}{\lambda +0.02\beta }- \frac{0.003}{{\beta }^{3}+1}$$

4 $$\lambda = \frac{R {\omega }_{T}}{{V}_{w}}$$

where ${P}_{turbine}$, ${C}_{p}$, $\rho$, $R$, ${V}_{w}$, $\lambda$, $\beta$, and ${\omega }_{T}$ are the captured power by wind turbine, power coefficient, air density, wind turbine blade radius, the speed of wind, tip speed ratio, pitch angle and wind turbine rotational speed, respectively. Figure 1 shows the variation of wind turbine power with rotational speed under several wind speeds, indicating that the wind turbine output power is affected by wind speed and its rotational speed^22,57^.

**Figure 2:**
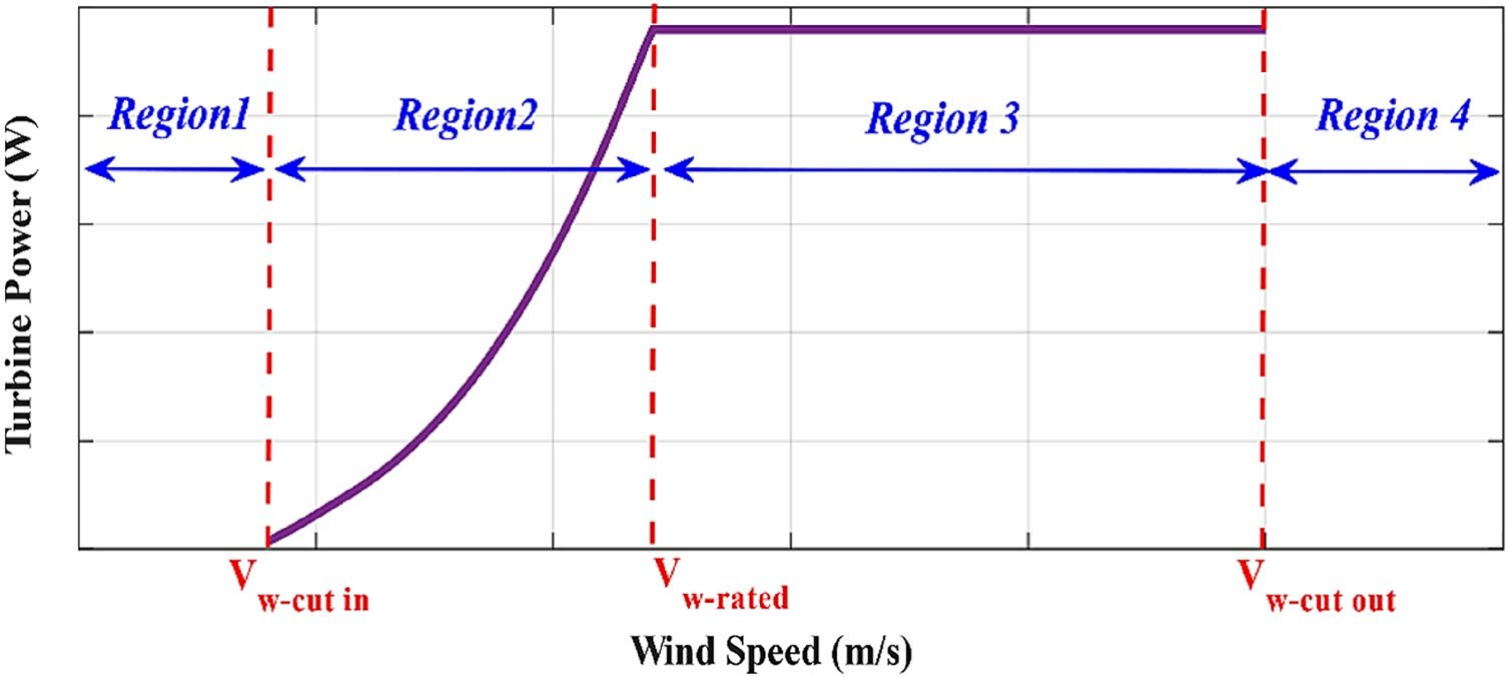
The wind turbine power with wind speed characteristic.

A wind turbine operates in four operational regions, as depicted in Fig. 2. The wind turbine does not generate power at wind speeds below a cut-in wind speed. The use of a maximum power point tracking technique in addition to the execution of suitable yaw and pitch angle control methods enables the wind turbine to produce the possible peak power when the wind speed is increased beyond the cut-in wind speed. A pitch angle controller is used to regulate the blades pitch angle when the wind speed exceeds the rated speed to limit the wind turbine power at its rated value for protecting wind turbine from damage. Wind turbine rotor braking is activated at wind speeds greater than the cut-out wind speed, stopping any further power generation for the wind turbine protection^58–60^.

## DFIG steady-state model

**Figure 3:**
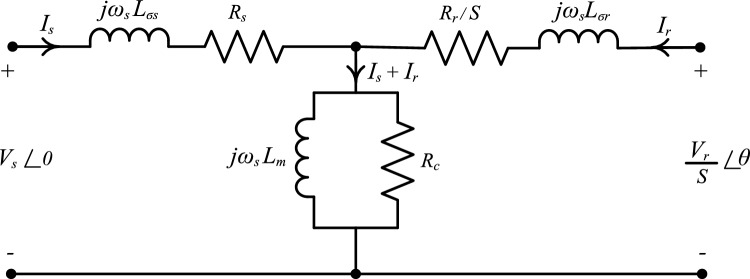
Equivalent circuit per phase of DFIG steady state model.

The DFIG steady-state electrical equivalent circuit can be ideally simplified, as shown in Fig. 3. The steady-state DFIG model takes iron losses into consideration. The positive direction is chosen as a motor. The vector of stator voltage is selected as a reference voltage with zero angle. *R*_*s*_*, R*_*r*_*, R*_*m*_ are phase stator resistance, phase rotor resistance referred to stator, and magnetizing resistance, respectively. ${L}_{\sigma s}$, ${L}_{\sigma r}$, ${L}_{m}$ are leakage stator inductance, leakage rotor inductance referred to stator, and magnetizing inductance, respectively. ${\omega }_{s}$, ${\omega }_{r}$, *S* are angular stator frequency, angular rotor frequency, and slip, respectively. *V*_*s*_,* V*_*r*_, $\theta$ are stator voltage magnitude, rotor voltage magnitude referred to stator, and rotor voltage angle, respectively. *I*_*s*_,* I*_*r*_, are stator current and referred rotor current, respectively^12,61–64^.

The steady-state equations of DFIG can be considered as follow. The stator and rotor currents equations can be considered as follow^65^:

5 $$ \begin{aligned} I_{s} & = \frac{1}{{\sigma L_{s} }} \{ [Fc_{s} \left| {V_{r} } \right|\cos (f + \theta ) + Fa_{r} \left| {V_{s} } \right|\cos (f + b_{r} )] \\ & \quad + j [Fc_{s} \left| {V_{r} } \right|\sin (f + \theta ) + Fa_{r} \left| {V_{s} } \right|\sin (f + b_{r} )]\} \\ & \quad - \frac{{L_{m} }}{{\sigma L_{s} L_{r} }} \{ [Fc_{r} \left| {V_{s} } \right|\cos (f) + Fa_{s} \left| {V_{r} } \right|\cos (f + b_{s} + \theta )]\} \\ & \quad + j[Fc_{r} \left| {V_{s} } \right|\sin (f) + Fa_{s} \left| {V_{r} } \right|\sin (f + b_{s} + \theta )] \} \\ \end{aligned} $$

6 $$ \begin{aligned} I_{r} & = \frac{{ - L_{m} }}{{\sigma L_{s} L_{r} }} \{ [Fc_{s} \left| {V_{r} } \right|\cos (f + \theta ) + Fa_{r} \left| {V_{s} } \right|\cos (f + b_{r} )] \\ & \quad + j [Fc_{s} \left| {V_{r} } \right|\sin (f + \theta ) + Fa_{r} \left| {V_{s} } \right|\sin (f + b_{r} )]\} \\ & \quad + \frac{1}{{\sigma L_{r} }} \{ [Fc_{r} \left| {V_{s} } \right|\cos (f) + Fa_{s} \left| {V_{r} } \right|\cos (f + b_{s} + \theta )]\} \\ & \quad + j\left[ {Fc_{r} \left| {V_{s} } \right|\sin (f) + Fa_{s} \left| {V_{r} } \right|\sin (f + b_{s} + \theta )} \right] \} \\ \end{aligned} $$

The active and reactive power of stator and rotor are^65,66^:

7 $$ \begin{aligned} P_{s} & = 3Real\left( {V_{s} I_{s}^{*} } \right) = 3 \left| {V_{s} } \right|^{ } \frac{M}{{\sigma L_{s} }} \{ c_{s} \left| {V_{r} } \right|\cos \left( {m + \theta } \right) + a_{r} \left| {V_{s} } \right|\cos \left( {m + b_{r} } \right) \\ & \quad - \frac{{L_{m} }}{{L_{r} }}\left[ {c_{r} \left| {V_{s} } \right|\cos \left( m \right) + a_{s} \left| {V_{r} } \right|\cos \left( {m + b_{r} + \theta } \right)} \right]\} \\ \end{aligned} $$

8 $$ \begin{aligned} Q_{s} & = 3Imag\left( {V_{s} I_{s}^{*} } \right) = 3 \left| {V_{s} } \right|\frac{M}{{\sigma L_{s} }}\{ - c_{s} \left| {V_{r} } \right|\sin \left( {m + \theta } \right) - a_{r} \left| {V_{s} } \right|\cos \left( {m + b_{r} } \right) \\ & \quad + \frac{{L_{m} }}{{L_{r} }}[c_{r} \left| {V_{s} } \right|\sin \left( m \right) + a_{s} |V_{r} |\sin \left( {m + b_{r} + \theta } \right]\} \\ \end{aligned} $$

9 $$ \begin{aligned} P_{r} & = 3Real\left( {V_{r} I_{r}^{*} } \right) = 3 \left| {V_{r} } \right|^{ } \frac{M}{{\sigma L_{r} }} \{ c_{r} \left| {V_{s} } \right|\cos \left( {m - \theta } \right) + a_{s} \left| {V_{r} } \right|\cos \left( {m + b_{s} } \right) \\ & \quad - \frac{{L_{m} }}{{L_{s} }}\left[ {c_{s} \left| {V_{r} } \right|\cos \left( m \right) + a_{r} \left| {V_{s} } \right|\cos \left( {m + b_{r} - \theta } \right)} \right]\} \\ \end{aligned} $$

10 $$ \begin{aligned} Q_{r} & = 3Imag\left( {V_{r} I_{r}^{*} } \right) = 3 \left| {V_{r} } \right|\frac{M}{{\sigma L_{r} }}\{ - c_{r} \left| {V_{s} } \right|\sin \left( { - m + \theta } \right) - a_{s} \left| {V_{r} } \right|\sin \left( {m + b_{s} } \right) \\ & \quad + \frac{{L_{m} }}{{L_{s} }}[c_{s} \left| {V_{r} } \right|\sin \left( m \right) - a_{r} |V_{s} |\sin \left( {m + b_{r} - \theta } \right]\} \\ \end{aligned} $$

where $${L}_{s}=$$ $${L}_{m}+ {L}_{\sigma s}$$, $${L}_{r}$$ = $${L}_{m}+ {L}_{\sigma r}$$, $$\sigma =1-\frac{{L}_{m}^{2}}{{L}_{s}{L}_{r}}$$, $${K}_{1}=\frac{{R}_{s}{R}_{r}}{\sigma {L}_{s}{L}_{r}}-{\omega }_{s}{\omega }_{r}$$, $${K}_{2}=\frac{{\omega }_{s}{R}_{r}}{\sigma {L}_{r}}+\frac{{\omega }_{r}{R}_{s}}{\sigma {L}_{s}}$$, $$m={tan}^{-1}(-\frac{{K}_{2}}{{K}_{1}})$$, M=$$\sqrt{\frac{1}{{K}_{1}^{2}+{K}_{2}^{2}}}$$, $${a}_{s}=\sqrt{\frac{{R}_{s}^{2}}{\sigma {L}_{s}}+{\omega }_{s}^{2}}$$, $${a}_{r}=\sqrt{{(\frac{{R}_{r}}{\sigma {L}_{r}})}^{2}+{\omega }_{r}^{2}}$$, $${b}_{s}={tan}^{-1}(\frac{\sigma {L}_{s}{\omega }_{s}}{{R}_{s}})$$, $${b}_{r}={tan}^{-1}(\frac{\sigma {L}_{r}{\omega }_{r}}{{R}_{r}})$$, $${c}_{s}=\frac{{R}_{s}{L}_{m}}{\sigma {L}_{s}{L}_{r}}$$, and $${c}_{r}=\frac{{R}_{r}{L}_{m}}{\sigma {L}_{s}{L}_{r}}$$

The DFIG mechanical power and power losses expressions are^65^:

11 $${P}_{mech}= {P}_{s}+ {P}_{r}-{P}_{ir}-{P}_{cu}^{s}-{P}_{cu}^{r}$$

12 $${P}_{cu}^{s}=3 {\left|{I}_{s}\right|}^{2} {R}_{s}$$

13 $${P}_{cu}^{r}=3 {\left|{I}_{r}\right|}^{2} {R}_{r}$$

14 $${P}_{ir}=3 \frac{{\left|{V}_{s}\angle 0- {I}_{s}({R}_{s}+j{\omega }_{s}{L}_{\sigma s})\right|}^{2}}{{R}_{c}}$$

## Bonobo optimizer

**Figure 4:**
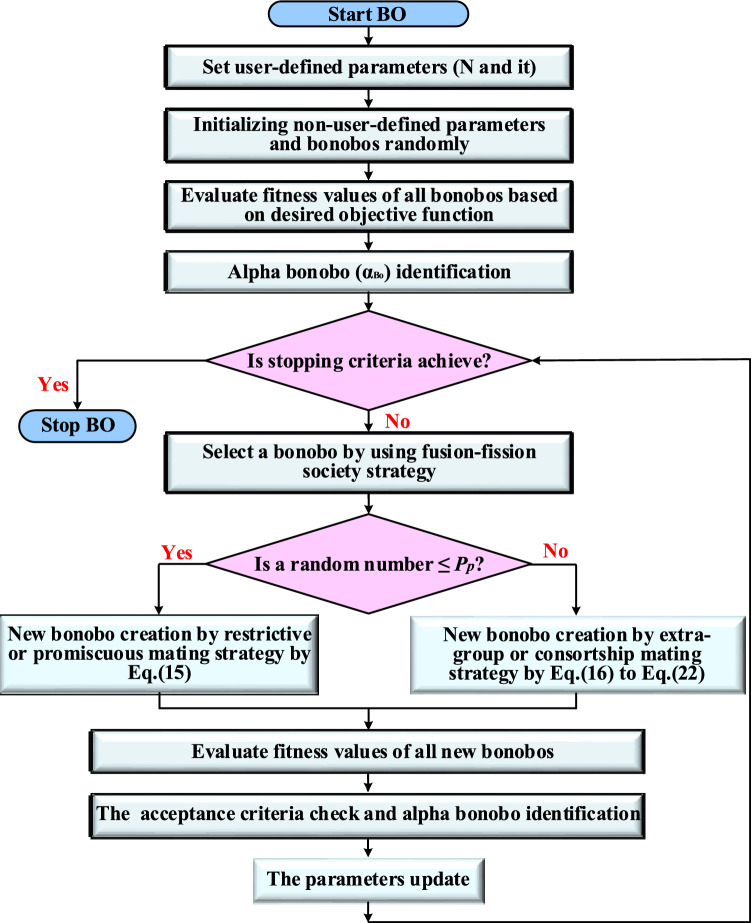
The flowchart of BO.

BO is one of the more recent intelligent heuristic optimization techniques. It is developed by Das and Pratihar^67^. It simulates numerous interesting aspects of the social behaviour and reproductive techniques of bonobos, often known as pygmy chimpanzees. Bonobos have a fission–fusion kind of social structure where, fission type occurs first, then the fusion type. For the fission type, they split off into a number of groups with various compositions and sizes and moving throughout the territory. For the fusion type, they merge again with the members of their community to carry out particular activities. To maintain a perfect social harmony, bonobos have four distinct methods of reproduction such as promiscuous mating, restrictive mating, consortship mating, and extra-group mating. The search technique with self-adjusting parameters is developed in such a way that it can efficiently cope with several states during solving various problems. Additionally, fission–fusion technique is a novel method in meta-heuristic algorithms that is used to choose the mating partner. These natural techniques are mathematically modelled in BO for solving an optimization problem, as shown in Fig. 4^68,69^.

Initially, BO includes two situations: positive situation and negative situation. The positive situation is best suited to peaceful living circumstances. On the other hand, a negative situation indicates that the absence of the aforementioned conditions for peaceful and well living. In each iteration, BO starts by initializing the parameters. Bo parameters are of two types, user-defined and non-user-defined parameters. The user-defined parameters are population size (N), iteration number (it). BO is an algorithm that operates with two population sizes; constant population size and random population initialization. While, the non-user-defined parameters of BO, such as phase probability (${P}_{p}$), extra-group mating probability (${P}_{xgm}$), positive phase count ($ppc$), negative phase count ($npc$), temporary sub-group size factor (${tsgs}_{factor}$), and directional probability (${P}_{d}$). Then, the objective values of all bonobos are estimated to identify the alpha bonobo (${\alpha }_{Bo}$) which is the best solution among all the bonobos in the population at its current state. While the stopping criteria is not achieved, another bonobo is selected using the fission–fusion social strategy of bonobos, participates in mating. The mating strategies adopted are to be different based on the situation type. The probability of either restrictive or promiscuous mating is higher in a positive situation. While, the probability of either extra-group or consortship is more for negative situation. To provide equal importance to both types of mating techniques in a particular situation, the value of ${P}_{d}$ is initially set to 0.5. However, its value is updated based on the phase count number and current situation. The value of ${P}_{p}$ is between 0.5 and 1 for positive situation. While, the value of ${P}_{p}$ is between 0 and 0.5 for negative situation. If a random number (*r*) lying in the range of (0, 1), is equal to or less than ${P}_{p}$, a new bonobo is created either by restrictive or promiscuous mating by Eq. (15):

15 $$ \begin{aligned} Bo\_new_{j} & = Bo_{j}^{i} + r_{1} \times scab \times \left( {\alpha_{Bo}^{j} - Bo_{j}^{i} } \right) \\ & \quad + \left( {1 - r_{1} } \right) \times scsb \times g \times \left( {Bo_{j}^{i} - Bo_{j}^{p} } \right) \\ \end{aligned} $$

where, ${Bo\_new}_{j}$ and ${\alpha }_{Bo}^{j}$ are the ${j}$th variables of the offspring and alpha bonobo, respectively. *j* changes from 1 to d, where d is the total number of variables for the given optimization problem. ${Bo}_{j}^{i}$ and ${Bo}_{j}^{p}$ represent the ${j}$th variable of the ${i}$th and ${p}$th-bonobo, respectively. ${r}_{1}$ is a random number generated in the range between 0 and 1. scsb and scab are sharing coefficients for chosen p^th^ bonobo and ${\alpha }_{Bo}$, respectively. $g$ takes two only values 1 or -1. If *r* is greater than or equal to ${P}_{p}$, a new bonobo is created through extra-group or consortship mating techniques using equations from Eqs. (16) to (22). If another random number (*r*_2_), in the range (0, 1), is either equal to or lower than ${P}_{xgm}$, a new bonobo is generated by the extra-group mating technique.

16 $${\tau }_{1}= {e}^{({r}_{4}^{2}+{r}_{4}-\frac{2}{{r}_{4}})}$$

17 $${\tau }_{2}= {e}^{\left(-{r}_{4}^{2}+2{r}_{4}-\frac{2}{{r}_{4}}\right)}$$

18 $$ Bo\_new_{j} = Bo_{j}^{i} + \tau_{1} \left( {Var\_max_{j} - Bo_{j}^{i} } \right)\quad if\quad \left( {\alpha_{Bo}^{j} \ge Bo_{j}^{i} \,and\, r_{3} \le p_{d} } \right) $$

19 $$ Bo\_new_{j} = Bo_{j}^{i} - \tau_{2} \left( {Bo_{j}^{i} - Var\_min_{j} } \right)\quad if\quad \left( {\alpha_{Bo}^{j} \ge Bo_{j}^{i} \,and\, r_{3} > p_{d} } \right) $$

20 $$ Bo\_new_{j} = Bo_{j}^{i} - \tau_{1} \left( {Bo_{j}^{i} - Var\_min_{j} } \right)\quad if\quad \left( {\alpha_{Bo}^{j} < Bo_{j}^{i} \,and\,r_{3} \le p_{d} } \right) $$

21 $$ Bo\_new_{j} = Bo_{j}^{i} + \tau_{2} \left( {Var\_max_{j} - Bo_{j}^{i} } \right)\quad if\quad \left( {\alpha_{Bo}^{j} \left\langle { Bo_{j}^{i} \,and\, r_{3} } \right\rangle p_{d} } \right) $$

22 $$ Bo\_new_{j} = \left\{ {\begin{array}{*{20}l} {Bo_{j}^{i} + g \times e^{{ - r_{5} }} \left( {Bo_{j}^{i} - Bo_{j}^{p} } \right)} \hfill & {if\,\, \left( {r_{2} > P_{xjm} \,\, and \,\,g = 1\, or\, r_{6} \le p_{d} } \right)} \hfill \\ {Bo_{j}^{p} } \hfill & {if\,\,(r_{2} > P_{xjm} )} \hfill \\ \end{array} } \right. $$

where ${\tau }_{1}$ and ${\tau }_{2}$ are the two intermediate measured values used for determining ${Bo\_new}_{j}$ value. *r*_*3*_ is a random number. ${r}_{4}$ is a random number between 0 and 1 and is not equal to 0. *r*_5_ and *r*_6_ are two random numbers between 0 and 1. ${Var\_min}_{j}$ and ${Var\_max}_{j}$ are the values of the lower and upper boundaries corresponding to the ${j}$th variable, respectively. Then, If the fitness value of the $Bo\_new$ is better than the ${Bo}^{i}$ or a random number lying between 0 and 1 is equal to or less than ${P}_{xgm}$, $Bo\_new$ is accepted. Additionally, ${Bo}^{i}$ replaces by the new one in the bonobo population. However, if $Bo\_new$ fitness value is found to be better as compared to that of the ${\alpha }_{Bo}$, the $Bo\_new$ is identified as the ${\alpha }_{Bo}$. Finally, if the ${\alpha }_{Bo}$ of current iteration has the better fitness value than that of the previous iteration, the parameters of BO are modified.

## Results and discussion

The wind turbine and DFIG parameters employed in this paper are illustrated in Table 1. The MATLAB software is used in this paper to develop an analytical steady-state model of the DFIG and wind turbine. The optimal magnitudes and angles of injected rotor voltage, wind turbine pitch angles, and maximum DFIG mechanical powers are obtained by three optimization techniques; BO, DTA, and PSO where the stator and rotor currents are less than its rated values during stator voltage dips from 0.2 ${V}_{s}^{rated}$ to 0.9 ${V}_{s}^{rated}$ with 0.1 step at all values of the wind speeds (4–25 m/s) with 0.1 step.

**Table 1:** DFIG and wind turbine parameters^22^.

**DFIG**	
Rated stator power	2 MW
Rated stator line voltage	690 V (rms)
Rated stator current	1760 A (rms)
Rated rotor current	1823 A (rms)
Rated rotor line voltage	845 V (rms)
Rated stator frequency	50 Hz
Rated rotor speed	1800 rpm
Rated rotor speed range	900–1800 rpm
Turn ratio (N_s_/Nr)	0.34
Pole number	4
R_s_, R_r_	2.6, 2.9 mΩ
L_s,_ L_r_	87, 87 μH
L_m_	2.5 mH
**Wind turbine**	
Rated mechanical power	2.4 MW
Rotor diameter	42 m
Wind speed range	4–25 m/s
Rated wind speed	12.1 m/s
Gearbox ratio	100
Nominal rotor speed range	9–19 rpm

The optimization objective function (${OF}_{1}$) is minimizing the sum of DFIG mechanical power and stator reactive power where their sign is negative at every wind speed, as illustrated in Eq. (23). The objective function is achieved by the variation of two input variables: the magnitude and angle of rotor voltage.

23 $${OF}_{1}=minimize\left({P}_{mech}+{Q}_{s}\right)+penality$$

**Figure 5:**
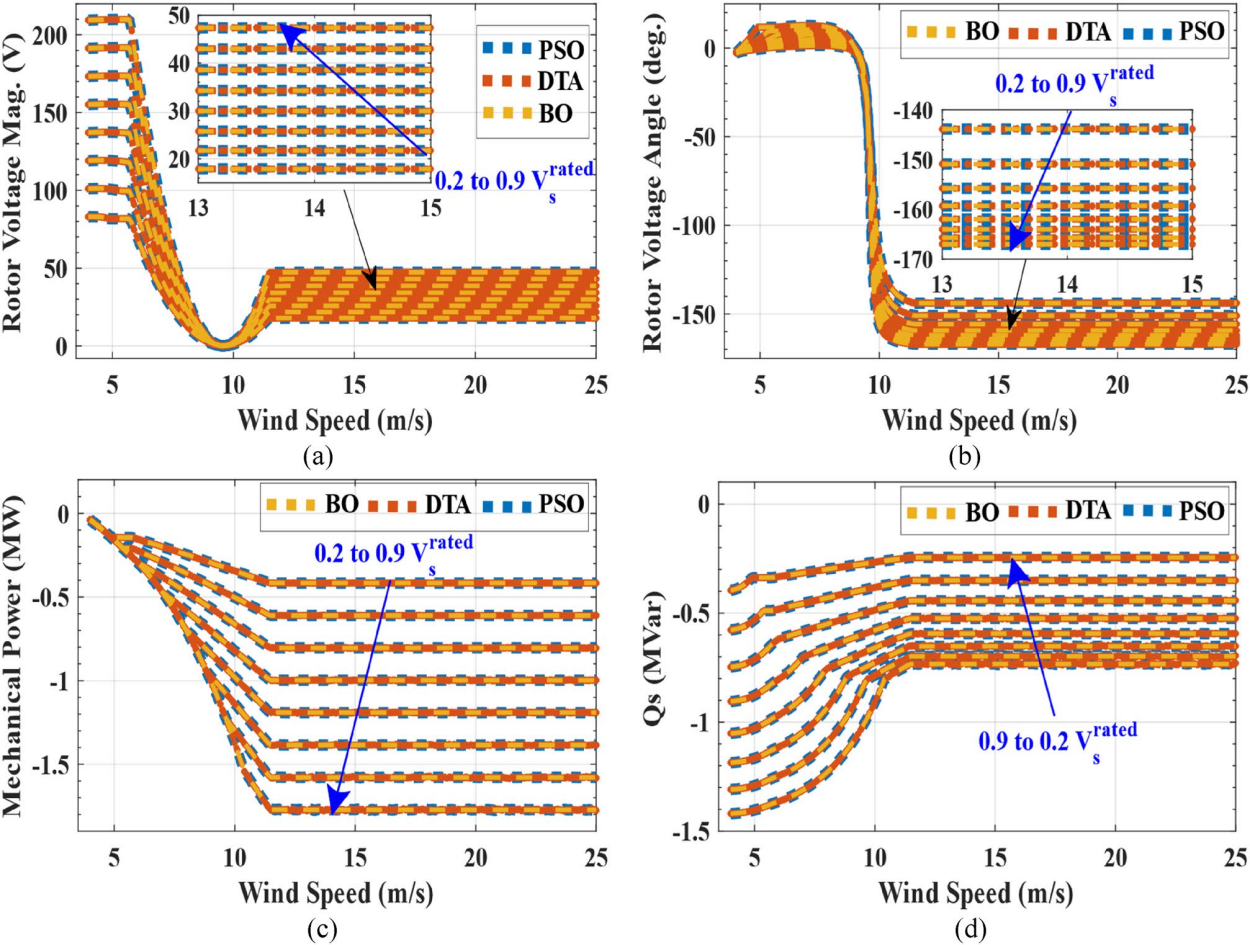
DFIG characteristics with wind speed at Vs = ${0.2V}_{s}^{rated}$ to ${0.9V}_{s}^{rated}$ (**a**) rotor voltage magnitude (**b**) rotor voltage angle (**c**) DFIG mechanical power (**d**) stator reactive power.

**Figure 6:**
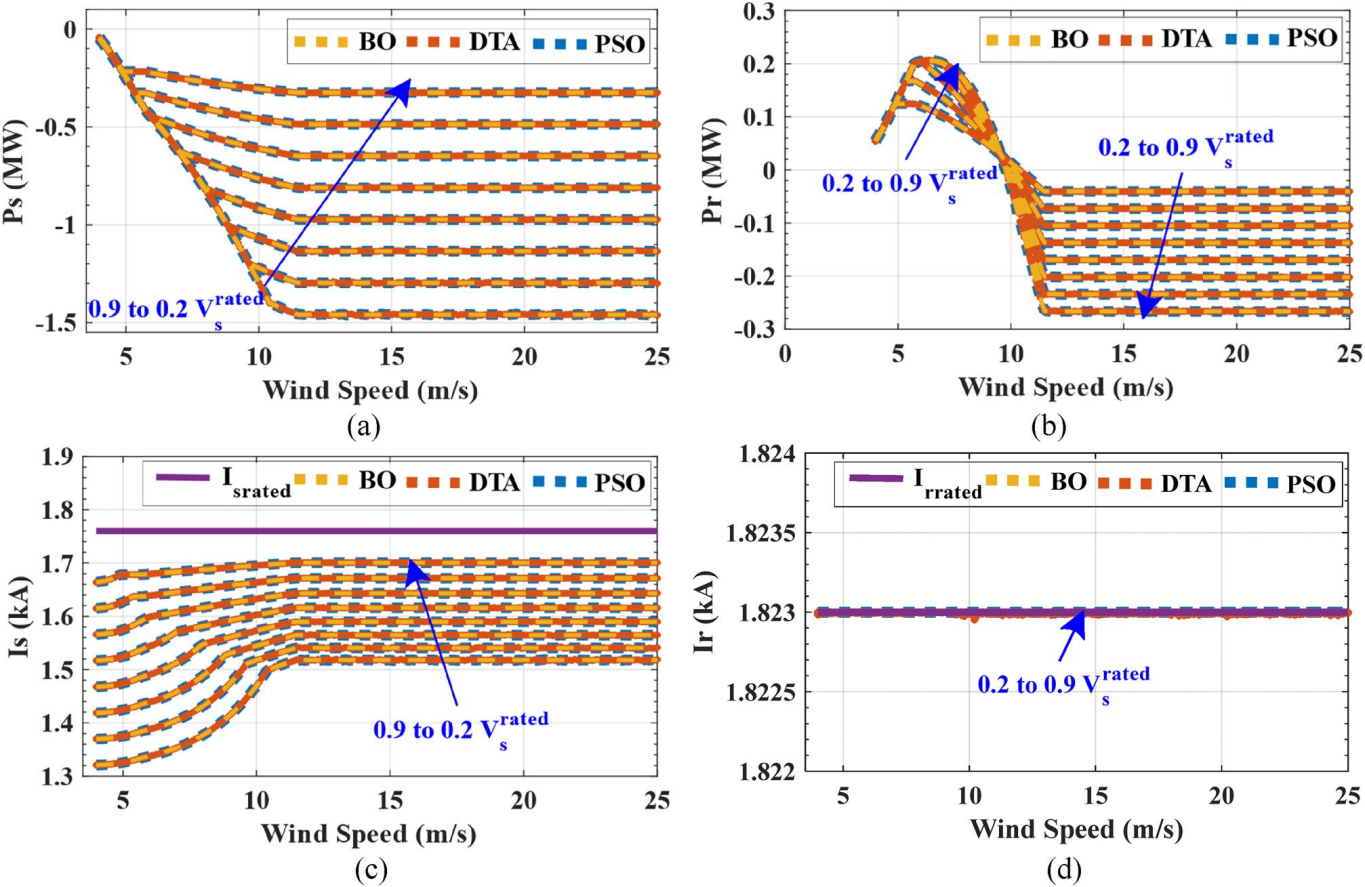
DFIG characteristics with wind speed at Vs = ${0.2V}_{s}^{rated}$ to ${0.9V}_{s}^{rated}$ (**a**) stator active power (**b**) rotor active power (**c**) stator current (**d**) rotor current.

**Figure 7:**
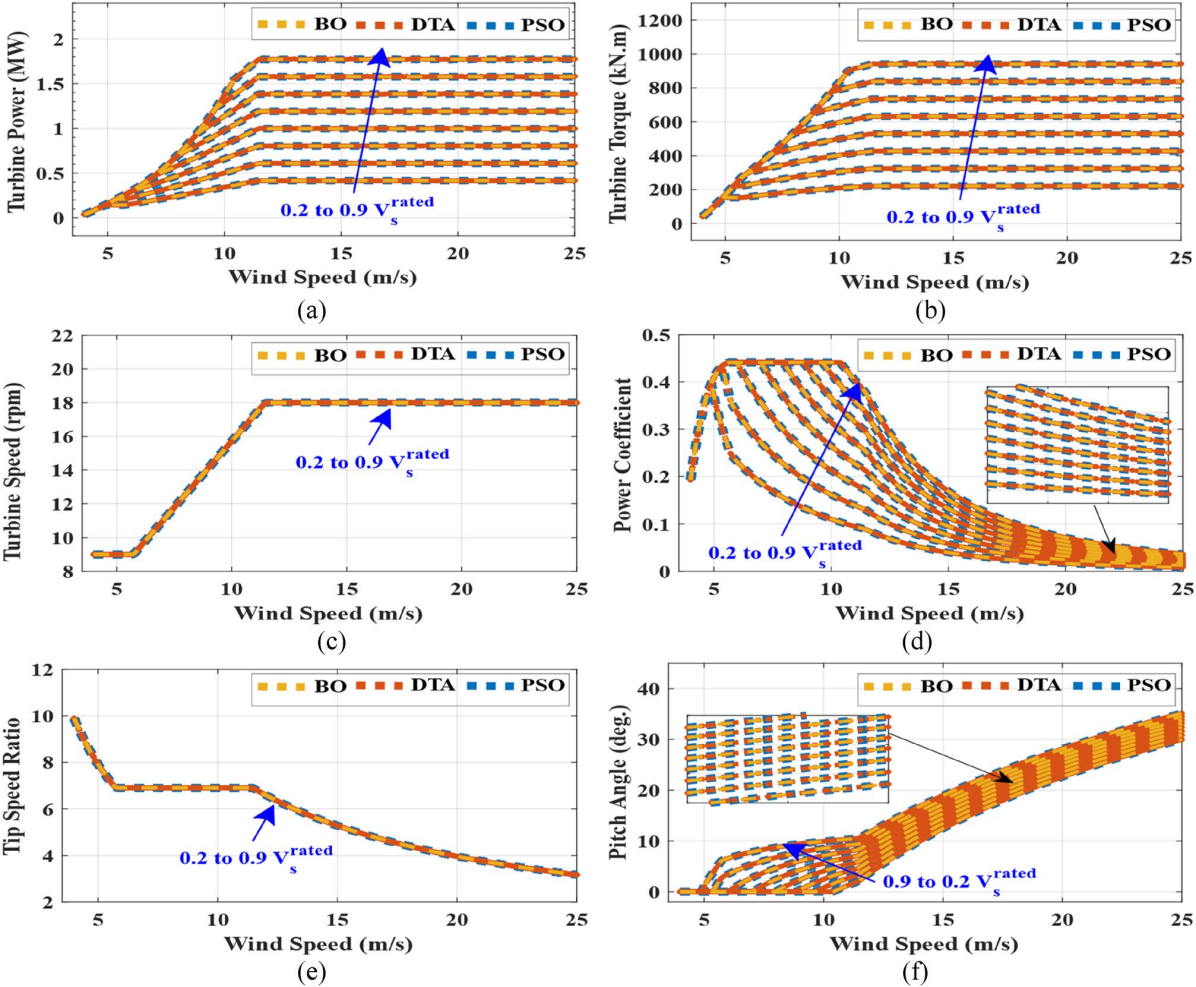
Wind turbine characteristics with wind speed at ${V}_{s}$ = ${0.2V}_{s}^{rated}$ to ${0.9V}_{s}^{rated}$ (**a**) wind turbine power (**b**) wind turbine torque (**c**) wind turbine rotational speed (**d**) power coefficient (**e**) tip speed ratio (**f**) pitch angle.

The penalty equals zero for feasible solutions. The unequal constraints of the objective function are depicted in Eq. (24).

24 $$ \left\{ {\begin{array}{*{20}c} {I_{s} \le I_{{s_{rated} }} } \\ {I_{r} \le I_{{r_{rated} }} } \\ {0 < P_{mech} < P_{t\_max} } \\ {Q_{s} < 0} \\ \end{array} } \right. $$

where ${P}_{mech}$ is DFIG mechanical power, ${I}_{{s}_{rated}}$ and ${I}_{{r}_{rated}}$ are the rated values of stator and rotor, respectively, and ${P}_{t\_max}$ is the maximum allowable wind turbine power at a certain wind speed. After obtaining maximum DFIG mechanical power which ensures that the stator and rotor currents do not exceed its rated values during stator voltage dips from 0.2 ${V}_{s}$ to 0.9 ${V}_{s}$ at all values of the wind speeds (4–25 m/s), three optimization methods are used to obtain the reference wind turbine pitch angles that achieve that wind turbine power is equal to the maximum DFIG mechanical power. The objective function that is used to obtain reference pitch angles at all wind speeds from 4 to 25 m/s is:

25 $${OF}_{2}=minimize\left(\left|{P}_{turbine}-{P}_{mech\_max}\right|\right)$$

where ${P}_{turbine}$ is wind turbine power and ${P}_{mech\_max}$ is maximum DFIG mechanical power which is obtained from ${OF}_{1}$. The results of three optimization algorithms are compared to guarantee the accuracy of the results. The optimal solution with the lowest objective function is selected after 50 trials using each of the three optimization strategies. The iterations number and population size are 200 and 1000 respectively for three optimization techniques. Table 2 shows the parameter settings for three optimization techniques. While, Table 3 depicts the statistics of results for three optimization techniques for ${OF}_{1}$ at 12 m/s wind speed in case of stator voltage dips to ${0.9V}_{s}^{rated}$. Table 4 depicts the statistics of results for three optimization techniques for ${OF}_{2}$ at 12 m/s wind speed and ${V}_{s}$=${0.9V}_{s}^{rated}$. Figures 5 and 6 show the characteristics of DFIG with wind speed in case of stator voltage dips from ${0.9V}_{s}^{rated}$ to ${0.2V}_{s}^{rated}$ over all wind speed range. Figure 7 presents the characteristics of wind turbine with wind speed in case of stator voltage dips from ${0.9V}_{s}^{rated}$ to ${0.2V}_{s}^{rated}$ over all wind speed range. Tables 5, 6 and 7 show comparisons between the DFIG characteristics for three optimization methods at ${V}_{w}$= 12 m/s and stator voltage changing from ${0.9V}_{s}^{rated}$ to ${0.2V}_{s}^{rated}$.

**Table 2:** Parameter settings for three optimization techniques.

*PSO*	
Inertia weight = 1	
Inertia weight damping ratio = 0.99	
Personal learning coefficient = 1.5	
Global learning coefficient = 2	
*DTA*	
Teaching factor = round (1 + rand)	
Where, rand is a random number from the interval [0,1]	
*BO*	
Initial probability for extra-group mating = 0.03	
Sharing coefficient for alpha bonobo = 1.25	
Sharing coefficient for selected bonobo = 1.3	
Rate of change in phase probability = 0.0035	
Maximum value temporary sub-group size factor = 0.05

**Table 3:** Optimization techniques statistics for ${OF}_{1}$ at ${V}_{W}$= 12 m/s and ${V}_{S}$ = 0.9 ${V}_{S}^{rated}$.

	PSO			DTA			BO		
	${OF}_{1}$ $\times {10}^{6}$	$\left|{V}_{r}\right|$	$\delta$	${OF}_{1}$ $\times {10}^{6}$	$\left|{V}_{r}\right|$	$\delta$	${OF}_{1}$ $\times {10}^{6}$	$\left|{V}_{r}\right|$	$\delta$
Min	− 2.5047	206.622	− 167.0119	− 2.5046	206.4275	− 167.0187	− 2.5047	206.7184	− 167.0052
Max	− 2.5047	206.757	− 166.9805	− 2.5045	206.7842	− 166.9355	− 2.5047	206.7281	− 167.0029
Mean	− 2.5047	206.710	− 167.0009	− 2.5046	206.6141	− 166.9790	2.5047	206.7230	− 167.0040
STD	0	0.0530	0.0124	0.00002	0.1459	0.0341	0	0.0035	0.0008

Friedman test	${OF}_{1}$	$\left|{V}_{r}\right|$	$\delta$						
Chi-Square distribution	10	0.4	0.4						
The degree of freedom	2	2	2						
Asymptotic significances	0.007	0.818731	0.818731

**Table 4:** Optimization techniques statistics for ${OF}_{2}$ at ${V}_{W}$ =12 m/s and ${V}_{S}$ = ${0.9V}_{S}^{rated}$.

	PSO		DTA		BO	
	${OF}_{2}$ $\times {10}^{-10}$	$\beta$	${OF}_{2}$ $\times {10}^{-10}$	$\beta$	${OF}_{2}$ $\times {10}^{-10}$	$\beta$
Min	4.6566	3.9290	2.3283	3.9211	2.3283	3.9224
Max	4.6566	3.9290	2.3283	3.9211	2.3283	3.9224
Mean	4.65661	3.9290	2.3283	3.9211	2.3283	3.9224
STD	0	0	0	0	0	0

Friedman test	${OF}_{2}$	$\beta$				
Chi-Square distribution	10	10				
The degree of freedom	2	2				
Asymptotic significances	0.007	0.007

**Table 5:** Comparison between DFIG characteristics based on PSO at ${V}_{W}$=12 m/s.

${V}_{s}/{V}_{s}^{rated}$	$\left|{V}_{r}\right|$ (V)	$\delta$ (°)	${P}_{m}$ (kW)	${Q}_{s}$ (kVar)	${I}_{s}$ (kA)	${I}_{r}$ (kA)	$\beta$ (°)
0.9	47.2145	− 167.0053	− 1.7713	− 0.7334	1.5177	1.8230	3.9234
0.8	42.8223	− 165.6644	− 1.5778	− 0.6986	1.5405	1.8230	5.1767
0.7	38.4580	− 164.0170	− 1.3845	− 0.6521	1.5646	1.8230	6.3888
0.6	34.1369	− 161.9554	− 1.1907	− 0.5942	1.5897	1.8230	7.5657
0.5	29.8708	− 159.2969	− 0.9971	− 0.5244	1.6159	1.8230	8.7058
0.4	25.6973	− 155.7830	− 0.8029	− 0.4436	1.6430	1.8230	9.8165
0.3	21.6439	− 150.8799	− 0.6100	− 0.3497	1.6715	1.8230	10.8892
0.2	17.8280	− 143.8496	− 0.4163	− 0.2449	1.7006	1.8230	11.9378

**Table 6:** Comparison between DFIG characteristics based on DTA at ${V}_{W}$=12 m/s.

${V}_{s}/{V}_{s}^{rated}$	$\left|{V}_{r}\right|$ (V)	$\delta$ (°)	${P}_{m}$ (kW)	${Q}_{s}$ (kVar)	${I}_{s}$ (kA)	${I}_{r}$ (kA)	$\beta$ (°)
0.9	47.1941	− 166.9847	− 1.7745	− 0.7301	1.5186	1.8230	3.9019
0.8	42.8372	− 165.6823	− 1.5756	− 0.7008	1.5399	1.8230	5.1909
0.7	38.4771	− 164.0436	− 1.3819	− 0.6546	1.5639	1.8230	6.4044
0.6	34.1463	− 161.972	− 1.1896	− 0.5953	1.5894	1.8230	7.5724
0.5	29.8747	− 159.3053	− 0.9967	− 0.5248	1.6158	1.8230	8.7081
0.4	25.6883	− 155.7580	− 0.8037	− 0.4428	1.6432	1.8230	9.8120
0.3	21.6481	− 150.8969	− 0.6097	− 0.3499	1.6714	1.8230	10.8909
0.2	17.8337	− 143.8859	− 0.4159	− 0.2453	1.7006	1.8230	11.9397

**Table 7:** Comparison between DFIG characteristics based on BO at ${V}_{W}$=12 m/s.

${V}_{s}/{V}_{s}^{rated}$	$\left|{V}_{r}\right|$ (V)	$\delta$ (°)	${P}_{m}$ (kW)	${Q}_{s}$ (kVar)	${I}_{s}$ (kA)	${I}_{r}$ (kA)	$\beta$ (°)
0.9	47.2137	− 167.0046	− 1.7714	− 0.7333	1.5178	1.8230	3.9226
0.8	42.8225	− 165.6646	− 1.5778	− 0.6987	1.5405	1.8230	5.1769
0.7	38.4599	− 164.0196	− 1.3842	− 0.6523	1.5645	1.8230	6.3904
0.6	34.1372	− 161.9560	− 1.1906	− 0.5943	1.5897	1.8230	7.5660
0.5	29.8714	− 159.2983	− 0.9970	− 0.5245	1.6159	1.8230	8.7062
0.4	25.6915	− 155.7670	− 0.8034	− 0.4430	1.6432	1.8230	9.8136
0.3	21.6466	− 150.8909	− 0.6098	− 0.3499	1.6714	1.8230	10.8903
0.2	17.8288	− 143.8543	− 0.4162	− 0.2450	1.7006	1.8230	11.9381

From the results, three optimization techniques give very close results, which proves that these solutions for pitch angles and rotor voltage values are the optimal solutions. These obtained optimal rotor voltages and pitch angles achieve two goals of this LVRT capability which guarantee that the rotor and stator currents do not exceed the rated current and provide maximum stator reactive power to support the grid voltage during voltage sags. Therefore, LVRT capability can be improved by using these reference pitch angles and rotor voltages in the DFIG converter controller. The results show that the rotor voltage magnitude decreases with increasing stator voltage dip and wind speed increase until the wind speed reaches to near the rated wind speed, the rotor voltage increases slightly then it is fixed at wind speeds that are greater than the rated wind speed. While, the rotor voltage angle is about from 0° to 12° for wind speeds that are lower than the rated value and is about from -144° to -167° for wind speeds that are higher than the rated value. The maximum DFIG mechanical power reduced to 1.7 MW instead of 2.4 MW in normal conditions due to the voltage sag. While the maximum stator power reduced to 1.45 MW instead of 2 MW. The stator reactive power decreases with increasing stator voltage dip for the all range of stator voltage dip and wind speeds. The rotor currents are constant at its rated value (1823 A) for the all range of stator voltage dip and wind speeds. While, the stator currents are lower than its rated value (1760 A). The range of pitch angle values is about from 0° to 35° for the all range of stator voltage dip and wind speeds.

**Figure 8:**
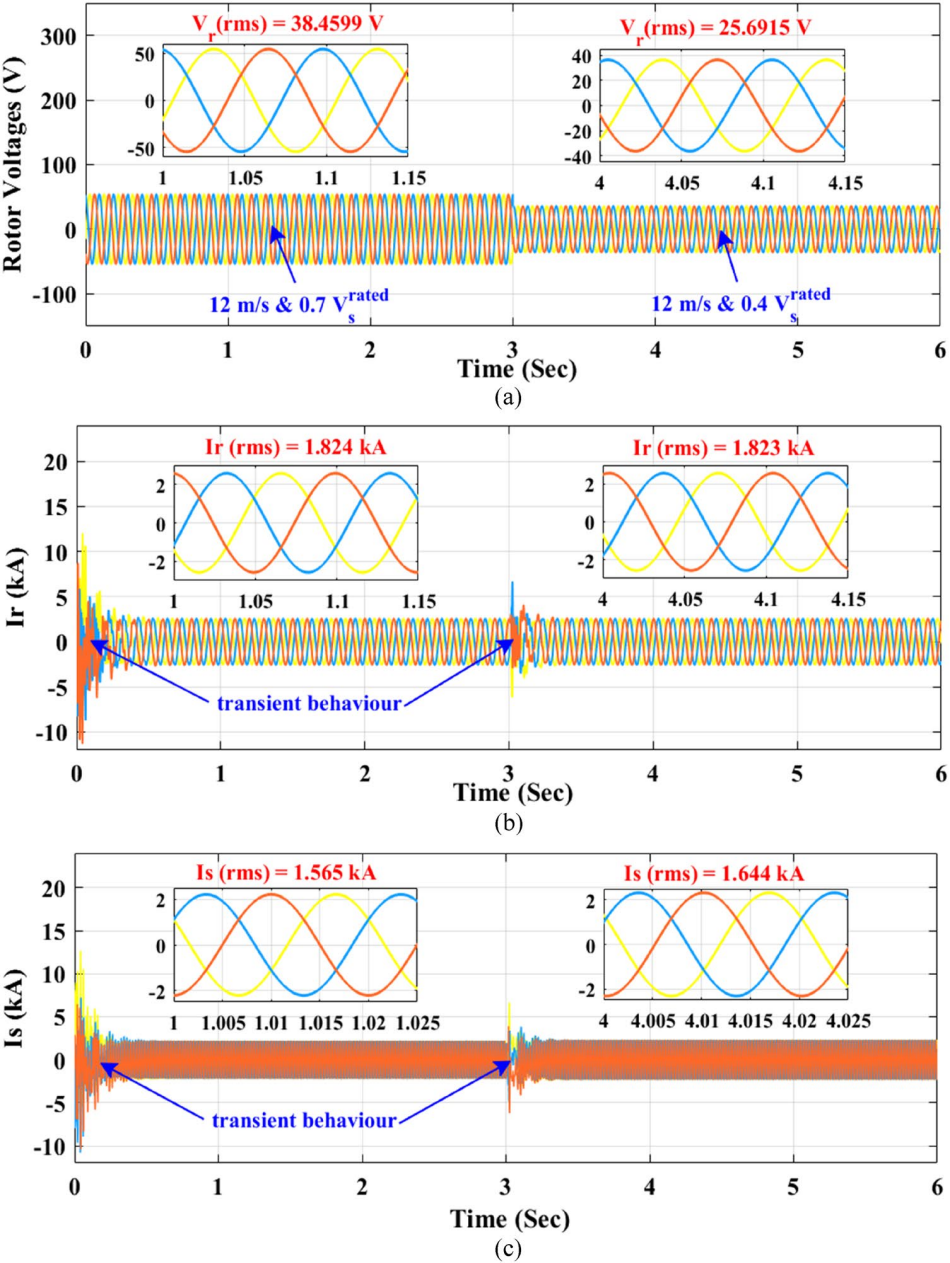
The DFIG waveforms at ${V}_{W}$=12 m/s, ${V}_{s}$ = ${0.7V}_{s}^{rated}$ and ${V}_{W}$=12 m/s, ${V}_{s}$ = ${0.4V}_{s}^{rated}$ (**a**) rotor voltages (**b**) rotor currents (**c**) stator currents.

To display the transient behaviour of the rotor voltage on the rotor currents and stator currents in response to the grid voltage dip, the dynamic model of DFIG in the Simulink-Matlab is used where the values of obtained rotor voltage from optimization techniques are injected to the rotor. Figure 8 shows the waveforms of rotor currents and stator currents at 12 m/s wind speed when stator voltage dips to ${0.7V}_{s}^{rated}$ and ${0.4V}_{s}^{rated}$. The waveforms of rotor currents and stator currents are reached to the steady-state values of currents which are obtained from optimization techniques.

### Human and animal rights

This article does not contain any studies with animals performed by any of the authors.

## ANFIS controller

The combination of neural network with fuzzy logic produced the ANFIS which is widely recommended for complicated problem solving and non-linear applications. This is because they can obtain the required performance by significantly changing the membership functions. Fuzzy logic provides the key principles of fuzzy set theory, fuzzy if–then rules, and approximate reasoning that address with information granularity and inaccuracy. Neural networks are capable of adapting and learning by regulating the interconnections between layers. The incorporation of this method is a two-level approach where the initial fuzzy model along with its input variables are derived with the use of the extracted rules from the data of input and output for a modelled system at the first level. Then, neural network is employed for fine tuning the initial fuzzy model rules in the next level which leads to the creation of the final ANFIS model of system^70–72^.

**Figure 9:**
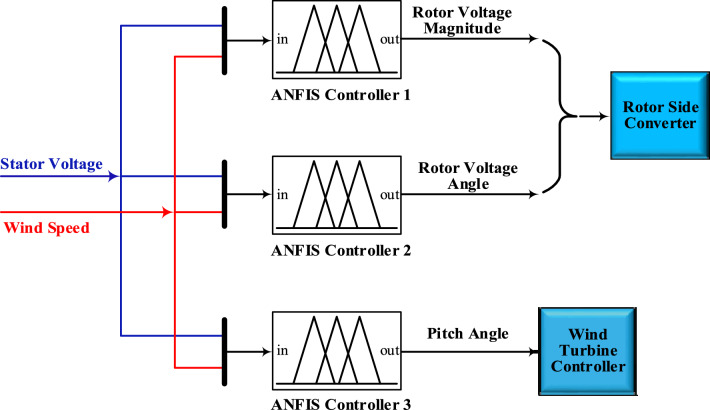
The proposed ANFIS configuration.

The major advantage of employing ANFIS in the proposed controller is its fast convergence time to meet with the varying wind speed or stator DFIG voltage. After obtaining the optimal magnitudes and angles of injected rotor voltage and wind turbine pitch angles by BO algorithm, these values are used to train the ANFIS controller. The proposed ANFIS configuration used is three ANFIS controllers because there are three outputs, as shown in Fig. 9. The output of first ANFIS controller is the magnitude value of injected DFIG rotor voltage. While, the output of second ANFIS controller is the angle value of injected DFIG rotor voltage and the output of third ANFIS controller is the pitch angle for wind turbine. The wind speed and stator DFIG voltage are inputs to three ANFIS controllers. Table 8 depicts the training performance for three ANFIS controllers. Table 9 shows the comparison between the results of the proposed ANFIS controller and BO algorithm at certain wind speeds and stator voltages that are not used in the training of ANFIS controllers. Results show that the proposed ANFIS controller and BO algorithm give very close results, and this proves that the effectiveness of the proposed ANFIS controller.

**Table 8:** The training performance of three ANFIS controllers.

	ANFIS Controller 1	ANFIS Controller 2	ANFIS Controller 3
Minimal training RMSE	0.000199	1.782957	0.211362
Epochs	10	1500	1500
Number of inputs MFs	1408	9	9
Inputs MFs type	Gauss	Gauss	Gauss
Output MF type	Linear	Linear	Linear

**Table 9:** Comparison between the proposed ANFIS controller and BO algorithm.

${V}_{s}$	${V}_{w}$	BO			ANFIS controllers		
		$\left|{V}_{r}\right|$ (V)	$\delta$ (°)	$\beta$ (°)	$\left|{V}_{r}\right|$ (V)	$\delta$ (°)	$\beta$ (°)
125	7.5	29.72	9.98	5.81	30.34	10.20	6.15
150	9	2.75	− 0.50	6.46	2.67	− 0.58	6.57
175	10.5	6.51	− 151.95	7.03	6.73	− 150.80	7.09
200	12	29.96	− 159.36	8.68	30.02	− 159.40	8.83
225	13.5	32.73	− 161.11	12.11	32.24	− 161.00	11.81
250	15	35.43	− 162.58	15.16	35.81	− 162.50	15.13
275	16.5	38.16	− 163.84	17.89	38.05	− 163.80	17.93
300	18	40.90	− 164.94	20.33	40.44	− 164.60	20.23
325	19.5	43.66	− 165.90	22.52	43.80	− 165.50	22.53
350	21	46.41	− 166.74	24.47	46.24	− 166.70	24.55

## Conclusion

In order to achieve LVRT capability, this paper introduced optimal reference magnitude and angle values of injected DFIG rotor voltage and reference pitch angles that provide the maximum possible DFIG mechanical power to ensure that rotor and stator currents do not exceed the rated values and also deliver maximum reactive power for supporting grid voltage during faults for all operating wind speeds. Recent optimization algorithm; BO was used to achieve the paper objective. To confirm the accuracy of results, the BO results were compared with two other optimization algorithms; PSO and DTA. Three optimization algorithms provided very close results, which proved that these solutions of rotor voltage values and pitch angles are the optimal solutions at any wind speed and any stator voltage dip. It is concluded that the rotor voltage magnitude generally decreases with increasing stator voltage dip where its range is about from 0 to 210 V for the all range of stator voltage dip and wind speeds. While, the rotor voltage angle is about from 12° to -167° and the range of pitch angle values is about from 0° to 35°. The maximum DFIG mechanical power reduced to 1.7 MW instead of 2.4 MW in normal condition due to the voltage sags. While the maximum stator power reduced to 1.45 MW instead of 2 MW. The stator and rotor currents do not exceed the rated values with the all range of stator voltage dip and wind speeds. ANFIS controller was employed as an adaptive controller for the prediction of the values of rotor voltage and wind turbine pitch angle for any stator voltage dip and any wind speed. The proposed controller with 2.4 MW wind turbine model and steady state model of DFIG considering iron losses was studied and simulated using the Matlab environment. The ANFIS controller achieved acceptable results as compared to BO results.

## Data Availability

The data that support the findings of this study are available from the corresponding author upon reasonable request.

## Abbreviations

WECS: Wind energy conversion system
DFIG: Doubly fed induction generator
LVRT: Low voltage ride through
BO: Bonobo optimizer
DTA: Driving training optimizer
PSO: Particle swarm optimizer
ANFIS: Adaptive neuro fuzzy inference system

## List of symbols

${P}_{turbine}$: Wind turbine captured power (W)
${C}_{p}$: Wind turbine power coefficient
$$\rho $$: Air density
$R$: Wind turbine blade radius (m)
$${V}_{w}$$: Wind speed (m/s)
${V}_{w\,cut\,in}$: Cut-in wind speed (m/s)
${V}_{w\,rated}$: Rated wind speed (m/s)
${V}_{w\,cut\,out}$: Cut-out wind speed (m/s)
$\lambda$: Tip speed ratio (rad)
$$\beta $$: Pitch angle (°)
${\omega }_{T}$: Wind turbine rotational speed (rad/s)
${R}_{s}$: Phase stator resistance ($\Omega$)
${R}_{r}$: Phase rotor resistance referred to stator ($\Omega$)
$${R}_{m}$$: Magnetizing resistance ($$\Omega $$)
${L}_{\sigma s}$: Leakage stator inductance (H)
${L}_{\sigma r}$: Leakage rotor inductance referred to stator (H)
$${L}_{m}$$: Magnetizing inductance (H)
${\omega }_{s}$: Angular stator frequency (rad/s)
${\omega }_{r}$: Angular rotor frequency (rad/s)
$$S$$: Slip
$$p$$: Pole number
${V}_{s}$: Stator voltage magnitude (V)
${V}_{r}$: Referred rotor voltage magnitude (V)
$\theta$: Rotor voltage angle (°)
$${I}_{s}$$: Stator current (A)
${I}_{r}$: Referred rotor current (A)
${P}_{s}$: Active stator power (W)
${Q}_{s}$: Reactive stator power (W)
${P}_{r}$: Active rotor power (W)
${Q}_{r}$: Reactive rotor power (W)
${P}_{mech}$: DFIG mechanical power (W)
$${P}_{ir}$$: Iron losses (W)
${P}_{cu}^{s}$: Stator copper losses (W)
${P}_{cu}^{r}$: Rotor copper losses (W)
